# Retroversion of the Pregnant Uterus

**Published:** 1868-07-15

**Authors:** R. G. Bogue

**Affiliations:** Surgeon to Cook County Hospital


### RETROVERSION OF THE PREGNANT UTERUS.

*Read before the Chicago Medical Society, by* R. G. BOGUE, M.D., *Surgeon to Cook County Hospital.*

OCTOBER 31st, 1866, was called to see Mrs. H., who asks relief for what she calls "falling of the womb," which has annoyed her very much of late.

*History*—She is of English birth, forty years of age, commenced menstruating at ten years, which has continued regular ever since, except when pregnant and nursing. Married at about twenty-three. Has had three or four children, the youngest twelve years ago. Soon after getting up from her last confinement, she began to have some distress in the pelvic region and back, which has continued more or less since then, at no time very severe. Her menstrual periods have passed off without much inconvenience until about three or four months ago, when they ceased. For a long time she has felt or noticed some fullness of the vulva after being upon her feet for some time, but don't recollect when she began to notice it; but during the last two and a half or three months it has been quite noticeable, and very markedly so for the last few weeks, with an increased amount of distress, and, aside from the pain in the back and hips, has had some pain along the thighs, but not severe; her chief annoyance now is from a "swelling," which protrudes from between the labia when upon her feet, and the increased suffering when in same position.

*Condition*—On examination, there was found presenting itself at the anterior part of the vulva a tumor, about the size of a hen's egg; soft, compressible, and not tender; which comes well out from between the labia while standing, but recedes when lying down. Pressing upon the posterior part of this tumor, and just within the vulva, was a hard body, which, she says, comes down even with the labia after standing or walking for some time. Pressing the finger upward along this for a little distance, there was a pretty short bend or flexion backward, terminating in a large globular tumor, filling up a good share of the hollow of the sacrum.

*Diagnosis*—Prolapse of the anterior wall of the vagina with posterior wall of the bladder, prolapse with flexion and curving forward toward the pubis of the cervix uteri, and retroversion of the uterus into the hollow of the sacrum. The cervix was long, but little if any enlarged, and not tender; the mouth was patulous, so that the end of the little finger could be introduced into it; there seemed to be no disease of the cervix nor of its canal.

The soft tumor was readily ascertained to be made up of the walls of the vagina and bladder, by introducing a sound into the bladder. There was no tenderness nor soreness of the parts; neither of the soft tumor, cervix, or body of the uterus.

The difficulty had been increasing quite rapidly during the last two months, but there had been no abrupt symptom which could mark the time of the displacement of the uterus. The cause of the enlargement of the womb certainly was not evident,—there being apparently no inflammatory condition of the organ, with cessation of the menses for the last three months, and an irritable condition of the mammæ for a time, it was certainly suspicious that she was pregnant, although there had been no morning nausea. On the other hand, could it not come from retained menses, they being confined within the cavity by the flexion of the neck?

*Queries in the case respecting treatment or interference*—One indication was, the restoration of the womb from its abnormal position. Effort was made to replace the organ by pressure upward with the fingers in the vagina, but without success.

The use of the sound would be proper if there were no pregnancy. If she were pregnant, she could not go on to full development with the organ in its present position; it might possibly, in course of development, rise up out of the pelvis, but this was only problematical; as it was, it could develop to a size quite filling the pelvis, then there would be, without doubt, abortion; and this with a good deal of risk to the patient, and probably after a great deal of suffering from pressure of the pelvic organs. My conviction was, that if she were pregnant, an abortion at that time, with the womb replaced, would be preferable to leaving the case to see what might become of it, or an abortion at a later time, when the womb could not be replaced. I therefore determined to try to pass the sound into the womb if it met with no obstruction at the internal os, but if it should, to solicit counsel in the case, not feeling willing to take the responsibility of producing an abortion.

After explaining the condition of things as I had found them to the patient, and giving her the general facts respecting her case, its probabilities, etc., I told her I would try to introduce the sound for the replacement of the womb; but if it met with any obstruction in its passage, I should stop, the more firmly believing her pregnant, being willing to do no more without counsel.

With the patient lying on her left side, knees drawn well up, and with the sound bent quite sharply about an inch from the end, I began its introduction. It passed along, without the least resistance, through the cervix into the cavity of the uterus, which was much enlarged, as shown from the depth the sound entered.

There was no pain from its introduction, nor from the efforts made for replacement. By gentle but persistent effort of lifting the organ upward, it was felt to move, when the direction of the sound could be changed from its face looking backward—as was necessary for introduction—to the face looking forward; and the back of the instrument, instead of pressing against the anterior commissure of the vulva, now pressed against the posterior one. The fundus uteri could now be felt through the abdominal walls, and upon vaginal examination the os could be felt in its normal position, looking downward and backward, instead of being found just beneath the symphisis pubis; and no globular tumor could be felt filling up the hollow of the sacrum. From all this there was left no doubt as to the diagnosis, nor of the replacement of the organ. Upon withdrawing the sound, it was found that it had entered the uterus to the depth of full five inches, and on it was fresh blood, leaving little or no doubt as to the cause of the enlargement of the organ, and from its malposition giving rise to the marked increase in the symptoms in the case.

She was directed to keep quiet, remaining recumbent the remainder of the day, and to be upon her feet as little as convenient, for a few days at least. She had some pain that evening, and the discharge of a small blood clot; but after that night she was comfortable; had less pain or distress in the pelvis and back, with less protrusion of the soft tumor.

She continued thus until November 23rd, thinking herself very comfortably over her trouble, believing herself not pregnant, and myself thinking it probable that the enlargement of the uterus to have been from some other cause; supposing it quite certain that an ovum would have been expelled soon, after having been jostled or injured by the introduction of the sound, with the manipulation necessary for the restoration of the uterus. But during the night of the 22nd November she began to have some pain in the back and in the pelvis, with a slight discharge of blood. I saw her in the morning; found her complaining as above. The pains were evidently uterine; the uterus could be felt above the brim of the pelvis; a few small blood clots had been expelled. Now it appeared that she was pregnant and about to abort.

I directed her to keep her bed for the day, and send me word if the pains should increase, or if there was much increase of the discharge. About eleven o'clock that evening I was called to her; there had been no marked increase in any of the symptoms until within an hour or two. I found her having quite severe labor pains, and upon vaginal examination, found the os dilated and the little fœtus presenting, which, with the placenta and membranes, was expelled in the course of a couple of hours.

Upon examination of the fœtus, there was found a slight bruise on one of the hips, which was the only mark to be found. On the placenta there was along one border, just within its attachment, a dark colored line of some two inches in length, narrow, a little hard, evidently colored by blood. This, I have no doubt, was caused by the uterine sound, the point where it passed separating the placenta from the uterine surface. The lady made a speedy and good recovery, and has been as well as before this pregnancy. She has yet the annoyance of the soft tumor or prolapsed vagina, but in a less degree than during it; but has not noticed the cervix at the vulva.

One question which suggests itself in the case, is the time when this retroversion occurred. This I could not learn. Another is, could the organ have been replaced by other means than that resorted to? I made effort only by use of the fingers in the vagina, which is, I believe, seldom of much value. I have seen notice since in some journal, of two cases of retroverted pregnant uteri being replaced by inflating a rubber bag in the vagina. This certainly would be a very harmless proceedure, and if effectual, the most simple. The colpeurynter could be used for this purpose; I should now make use of it in a similar case. Another, was the introduction of the sound justifiable under the circumstances, before other means had been tried? I did in the case what I thought at the time right and proper, yet felt not a little chagrined to find that I had been the means directly of procuring an abortion.

